# The Effects of Lead Exposure on Serum Uric Acid and Hyperuricemia in Chinese Adults: A Cross-Sectional Study

**DOI:** 10.3390/ijerph120809672

**Published:** 2015-08-18

**Authors:** Haijiang Dai, Zhijun Huang, Qihong Deng, Ying Li, Ting Xiao, Xingping Ning, Yao Lu, Hong Yuan

**Affiliations:** 1 The Third Xiangya Hospital, Central South University, Changsha 410013, China; E-Mails: daihaijiang1217@163.com (H.D.); mhzjxy3@163.com (Z.H.); lydia0312_2000@163.com (Y.L.); cwxyxiaoting@126.com (T.X.); 2 Institute of Environment and Health, Central South University, Changsha 410013, China; E-Mail: qhdeng@mail.csu.edu.cn; 3 Centers for Disease Control and Prevention, Zixing 423400, China; E-Mail: ningxp01@sina.com

**Keywords:** lead exposure, uric acid, hyperuricemia, non-occupational exposure

## Abstract

The aim of this study was to assess the correlation between blood lead levels and both serum uric acid and hyperuricemia in adult residents living within an area of China with lead pollution.  We conducted a cross-sectional analysis of 2120 subjects (1180 of whom were male) between the ages of 20 and 75 years who had undergone health examinations at the Centers for Disease Control and Prevention (CDC) in a lead-polluted area of China between January 2013 and August 2014. Blood lead was positively correlated with serum uric acid in both males (r = 0.095, *p* = 0.001) and females (r = 0.134, *p* < 0.001). Multivariate linear regression analysis demonstrated that for males, blood lead (*p* = 0.006), age (*p* = 0.001), current smoking (*p* = 0.012), education (*p* = 0.001), triglycerides (TG) (*p* < 0.001), and serum creatinine (*p* < 0.001) were independently associated with serum uric acid. For females, blood lead (*p* < 0.001), body mass index (BMI) (*p* = 0.009), and TG (*p* < 0.001) were independently associated with serum uric acid. After multiple adjustments, blood lead was significantly associated with a higher prevalence of hyperuricemia when female subjects were categorized into quartiles (for the highest quartile *vs.* the lowest quartile, odds ratio (OR) = 2.190; 95% confidence interval (CI): 1.106–4.338; *p* = 0.025); however, no such association was observed for male subjects. Continuous lead exposure has an independent impact on serum uric acid for both males and females, although this impact is more pronounced for females than for males. Lead exposure is significantly associated with hyperuricemia for females but not for males.

## 1. Introduction

Uric acid is the end-product of endogenous and dietary purine metabolism in humans. It is formed by the liver and mainly excreted by the kidneys (65%–75%) and intestines (25%–35%) [1]. Any factors that caused either higher synthesis or lower excretion of uric acid can make serum uric acid levels dramatically increase. Great blood cell depletion [1], the consumption of purine-rich foods [2], obesity [3], and renal diseases [4] are the common factors. Increasing evidence has demonstrated that mild to moderate increase in the serum uric acid levels, even within the normal range, is considered as a risk factor for cardiovascular diseases [5,6]. Hyperuricemia represents the abnormal increase of serum uric acid. Plenty of evidence suggests that hyperuricemia is a prerequisite for gout and is also associated with renal calculi, metabolic syndrome, cardiovascular events and all causes of mortality [7,8,9,10].

With the rapid industrial development that has occurred in China, lead pollution has become a serious problem. According to the report from lancet, the disability-adjusted life-years (DALYs) caused by lead exposure has increased 155% over the past 20 years [11]. Because industrial lead emissions are still rising and difficult to degrade, it will have an extremely long-term health effects on the Chinese people. Thus, it is important for researchers to study and understand the consequences of continuous lead exposure. High levels of serum uric acid have been reported associated with lead exposure in some studies [12,13,14]. However, some other studies have an objection to this result [15,16]. The aim of the present investigation was to evaluate the impact of blood lead on serum uric acid and hyperuricemia in a population-based sample of Chinese adults suffering continuous lead exposure.

## 2. Methods 

### 2.1. Study Population

This cross-sectional study was placed in Zixing city, which is very famous for its coal mining and lead mining. As a consequence of mining that lasted for several decades, significant amounts of lead have been released into the environment, causing an increase of blood lead level among the area’s local residents. We investigated 4324 residents living in Zixing city through cooperation with the Centers for Disease Control (CDC) of Zixing during June 2013 to September 2014. Among these participants, people between 20 and 75 years old were included in this study. The exclusion criteria were as follows: (1) the absence of data pertaining to age, gender, smoking, drinking, education, body weight, height, blood lead, uric acid, blood pressure, fasting glucose, total cholesterol (TC), triglyceride (TG), high-density lipoprotein cholesterol (HDL-C), serum creatinine, or blood urea nitrogen (BUN); (2) a history of malignancy, severe cardiovascular diseases, stroke, liver diseases, and pregnant females; (3) currently had kidney autoimmune diseases or infectious diseases or currently exposed to other known renal toxicants; (4) who were taking medications such as uric acid-lowering agents; and (5) duplicate cases. A total of 2120 eligible subjects were enrolled in this study. 

The Ethics Committee of the Third Xiangya Hospital approved this study and informed consent was obtained from each participant enrolled in this study.

### 2.2. Baseline Data Collection

Baseline information on lifestyle and demographic characteristics encompassing age, gender, education, medical history, smoking and drinking habits were obtained by a standardized questionnaire. Smoking was categorized as current smoking or nonsmokers (including never before smoked or ex-smokers). Alcohol consumption was categorized as current (at least once per week) or never. A three-level education variable was created on the basis of the number of years of education reported by the participants (0–9 years ≤ High school; 10–12 years = High school; and 13 years ≥ High school).

### 2.3. Anthropometric Measurements

Body height and weight were measured by the specially assigned nurses. Body mass index (BMI) was calculated as the participant's body weight (kg) divided by the square of the participant’s height (m^2^). After resting for 30 min, systolic and diastolic blood pressure (SBP and DBP) were measured twice in the participants’ right arms using a mercury sphygmomanometer. There was a 3-min interval between the two measurements for each participant, and the mean value of the two measurements was used for subsequent analyses.

### 2.4. Blood Lead Measurements

Fasting blood samples were collected by venipuncture from all participants with 5 mL of vacuum EDTA anticoagulant blood tubes. All blood samples were stored at −80 °C until laboratory analysis. The blood lead concentration was measured via graphite furnace atomic absorption spectrophotometry at the laboratories of the Zixing CDC. The standard of lead was prepared by using its standard solution (NRCCRM, China). Blood lead levels were expressed in μg/L. The limit of detection for total blood lead was 0.5 μg/L, and no participants exhibited blood lead values lower than the detection limit. Each sample was analyzed in duplicate, and the mean of both measurements was used for subsequent analyses. Participants were divided into quartiles according to blood lead levels: Quartile 1 (≤50.2 μg/L), Quartile 2 (>50.2 and ≤79.0 μg/L), Quartile 3 (>79.0 and≤126.0 μg/L), Quartile 4 (>126.0 μg/L).

### 2.5. Serum Uric Acid and Hyperuricemia

Serum uric acid levels were measured by the uricase method using a Hitachi 7020 Automatic Analyzer (Hitachi, Tokyo, Japan). Hyperuricemia was defined as serum uric acid levels ≥416 μmol/L (7.0 mg/dL) in male participants and ≥357 μmol/L (6.0 mg/dL) in female participants, similar to criteria used in previous studies [17,18]. 

### 2.6. Other Biochemical Measurements

After a ≥10 hours fast, serum glucose, TC, TG, and HDL-C levels were measured via enzymatic procedures using a Hitachi 7020 Automatic Analyzer (Hitachi, Tokyo, Japan). Serum creatinine was measured using Jaffe’s kinetic method and BUN was determined using a commercial kit according to manufacturer’s instructions in the central laboratory of Zixing CDC.

### 2.7. Statistical Analyses

The statistical analyses were performed using SPSS 22.0. Normally distributed data were expressed as means ± SD, whereas variables with a skewed distribution were reported as medians (interquartile range) and log transformed to approximate normality before analysis. Categorical variables are presented as percentages. Gender differences between quantitative variables were tested by Student’s *t* test or the Mann-Whitney U test. The chi-square test was used to test for differences in qualitative variables between genders. Pearson or Spearman rank correlation analyses were performed to examine the association between uric acid and various parameters. Multiple linear regression analyses were performed to identify any independent associations between uric acid and blood lead levels. The odds ratios (95% confidence interval) for hyperuricemia were calculated using a multiple logistic regression analysis after adjusting for confounding factors. *p* < 0.05 were considered statistically significant.

## 3. Results

### 3.1. General Characteristics of Participants

A total number of 2120 participants (1180 males and 940 females; median age: 44.0 years, range: 20−75 years) living in a region of China with lead pollution were included. The participants’ median blood lead concentration was 79 (50.2−126) μg/L, and their mean uric acid level was 289.4 ± 72.7 μmol/L. The prevalence of hyperuricemia in this region was 7.1%. Table 1 depicts the demographic, anthropometric, and biochemical characteristics of the 2120 participants by gender. The percentages of current smoking, drinking, and the BMI, SBP, DBP, TG, serum creatinine, uric acid, blood lead levels were significantly higher in males than in females (*p* < 0.05); however, the females were older and had a higher TC level than males. The percentages of education and hyperuricemia and the fasting glucose, HDL-C, BUN levels were not different between the genders.

**Table 1:** Baseline characteristics of the study participants.

Variables	Males (n = 1180)	Females (n = 940)	*p*
Age (years)	43.0 (35.0–48.0)	47.0 (38.0–58.0)	<0.001
BMI (kg/m^2^)	23.3 ± 3.1	22.6 ± 3.1	<0.001
Current smoking, n (%)	571 (48.4%)	44 (4.7%)	<0.001
Current drinking, n (%)	775 (65.7%)	183 (19.5%)	<0.001
Education, n (%)			0.412
>High school	103 (8.7%)	91 (9.7%)	
=High school	435 (36.9%)	364 (38.7%)	
<High school	642 (54.4%)	485 (51.6%)	
SBP (mmHg)	121.0 (112.0–130.0)	120.0 (108.0–130.0)	<0.001
DBP (mmHg)	81.3 ± 9.9	77.7 ± 9.7	<0.001
Fasting glucose (mmol/L)	4.6 (4.0–4.8)	4.5 (4.1–4.9)	0.074
TC (mmol/L)	4.1 (3.8–4.5)	4.2 (3.8–4.5)	0.003
TG (mmol/L)	1.4 (1.0–1.6)	1.3 (1.0–1.6)	0.034
HDL-C (mmol/L)	1.6 ± 0.2	1.6 ± 0.2	0.468
Serum creatinine (μmol/L)	89.3 ± 11.5	88.0 ± 7.4	0.004
BUN (mmol/L)	4.3 ± 0.9	4.4 ± 1.0	0.067
Uric acid (μmol/L)	311.6 ± 72.3	261.6 ± 63.1	<0.001
Hyperuricemia, n (%)	74 (6.3%)	76 (8.1%)	0.106
Blood lead (µg/L)	91.0 (58.0–149.0)	66.0 (44.0–103.0)	<0.001

Notes: BMI = body mass index, SBP = systolic blood pressure, DBP = diastolic blood pressure, TC = total cholesterol, TG = triglycerides, HDL-C = high-density lipoprotein cholesterol, BUN = blood urea nitrogen.

### 3.2. Correlations between Serum Uric Acid and other Variables

Serum uric acid level was positively correlated with blood lead (r = 0.095, *p* = 0.001 in males; r = 0.134, *p* < 0.001 in females), age, BMI, current smoking, SBP, and TG in both males and females (Table 2). The serum uric acid level was also positively correlated with education, serum creatinine, and BUN in males and positively correlated with DBP, TC in females but was not correlated with current drinking, fasting glucose, or HDL-C in either gender.

**Table 2:** Correlations between serum uric acid and other clinical parameters.

Variables	Males *r*	*p*	Females *r*	*p*
Blood lead (µg/L) *	0.095	0.001	0.134	<0.001
Age (years) *	0.127	<0.001	0.111	0.001
BMI (kg/m^2^)	0.089	0.002	0.129	<0.001
Current smoking	0.096	0.028	0.066	0.043
Current drinking	0.056	0.054	0.000	0.994
Education	0.121	0.001	−0.036	0.272
SBP (mmHg) *	0.075	0.010	0.083	0.011
DBP (mmHg)	0.048	0.097	0.082	0.012
Fasting glucose (mmol/L) *	0.054	0.063	0.056	0.088
TC (mmol/L) *	0.041	0.156	0.071	0.029
TG (mmol/L) *	0.165	<0.001	0.159	<0.001
HDL-C (mmol/L)	−0.056	0.054	−0.017	0.612
Serum creatinine (μmol/L)	0.281	<0.001	0.044	0.175
BUN (mmol/L)	0.179	<0.001	0.041	0.206

Notes: * represent log transformed variable. BMI = body mass index, SBP = systolic blood pressure, DBP = diastolic blood pressure, TC =total cholesterol, TG = triglycerides, HDL-C = high-density lipoprotein cholesterol, BUN = blood urea nitrogen.

### 3.3 Multiple Linear Regression Analysis for Serum Uric Acid Level

In a multiple linear regression analysis with all relevant variables included, log blood lead was a statistically significant variable in both males (β = 18.575, *p* = 0.006) and females (β = 27.374, *p* < 0.001) after the adjustment of related factors (age, BMI, current smoking, drinking, education, SBP, DBP, fasting glucose, TC, TG, HDL-C, creatinine, BUN). Other significant variables were shown in Table 3.

**Table 3:** Multiple linear regression analysis of the effects of independent variables on serum uric acid.

Variables	Males β	*p*	Females β	*p*
Blood lead *	18.575	0.006	27.374	<0.001
Age *	65.165	0.001	27.300	0.191
BMI	0.783	0.254	1.831	0.009
Current smoking	10.350	0.012	17.232	0.075
Current drinking	0.731	0.866	−3.294	0.531
Education	11.099	0.001	3.257	0.311
SBP *	13.244	0.834	−37.617	0.505
DBP	−0.022	0.944	0.296	0.370
Fasting glucose *	5.234	0.854	−2.509	0.924
TC *	−34.085	0.296	34.864	0.314
TG *	53.158	<0.001	43.553	<0.001
HDL-C	1.844	0.869	9.713	0.362
Serum creatinine	1.513	<0.001	0.108	0.700
BUN	3.125	0.198	1.274	0.544

Notes: * represent log transformed variable. BMI = body mass index, SBP = systolic blood pressure, DBP = diastolic blood pressure, TC =total cholesterol, TG = triglycerides. HDL-C = high-density lipoprotein cholesterol, BUN = blood urea nitrogen.

### 3.4. Association between Blood Lead Levels and the Prevalence of Hyperuricemia

**Figure 1:**
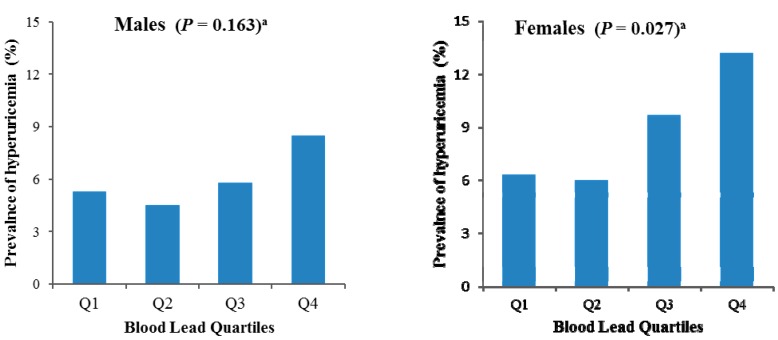
Unadjusted prevalences of hyperuricemia by blood lead quartiles for males and females. ^a^ *p* values determined by chi-square tests across the four quartiles.

When we assessed the association between blood lead levels and hyperuricemia as a categorical variable, we found that significant association between blood lead quartiles and unadjusted prevalence of hyperuricemia only in females (*p* = 0.027, Figure 1). In a multiple logistic regression analysis adjusted for age, BMI, current smoking, drinking, and education (Table 4; model 2), blood lead levels were positively associated with a higher prevalence of hyperuricemia when categorized in quartiles (the highest *vs.* the lowest quartile; OR, 2.249; 95% CI, 1.151–4.394; *p* = 0.018) in females. Further adjusting model 2 for SBP, DBP, fasting glucose, TC, TG, HDL-C, serum creatinine, BUN (Table 4; model 3), blood lead levels were still positively associated with a higher prevalence of hyperuricemia (the highest *vs.* the lowest quartile; OR, 2.190; 95% CI, 1.106–4.338; *p* = 0.025). However, there was no significant association between blood lead levels and the prevalence of hyperuricemia in males.

**Table 4:** Odds ratios (with 95% confidence intervals) for hyperuricemia by blood lead concentration.

Blood Lead (µg/L)	C/NC	Model 1	Model 2	Model 3
**Males**				
Quartile 1 (≤50.2)	12/214	1.00 (Ref)	1.00 (Ref)	1.00 (Ref)
Quartile 2(>50.2 and ≤79.0)	12/255	0.859 (0.364, 2.024)	0.953 (0.399, 2.275)	1.255 (0.480, 3.280)
Quartile 3(>79.0 and ≤126.0)	18/293	0.919 (0.416, 2.029)	0.917 (0.410, 2.053)	1.181 (0.485, 2.874)
Quartile 4 (>126.0)	32/344	1.508 (0.732, 3.106)	1.551 (0.746, 3.226)	1.708 (0.742, 3.933)
**Females**				
Quartile 1 (≤50.2)	19/285	1.00 (Ref)	1.00 (Ref)	1.00 (Ref)
Quartile 2(>50.2 and ≤79.0)	16/252	0.940 (0.472, 1.869)	0.950(0.475, 1.898)	0.928 (0.462, 1.863)
Quartile 3(>79.0 and ≤126.0)	21/196	1.516 (0.791, 2.905)	1.458(0.755, 2.815)	1.398 (0.716, 2.732)
Quartile 4 (>126.0)	20/131	2.294 (1.181, 4.456)	2.249(1.151, 4.394)	2.190 (1.106, 4.338)

C: cases; NC: non-cases; Model 1: Adjusted for age; Model 2: Further adjusted for current smoking, current drinking, education, and body mass index; Model 3: Further adjusted for systolic blood pressure, diastolic blood pressure, fasting glucose, total cholesterol, triglycerides, high-density lipoprotein cholesterol, serum creatinine, and blood urea nitrogen.

## 4. Discussion

In this cross-sectional study, we found that blood lead was associated with serum uric acid in local male and female residents. In females, participants in the highest blood lead quartiles had significantly higher prevalence of hyperuricemia compared to the lowest blood lead quartile. In addition, all of these associations remained statistically significant after adjusting for confounding factors, which were known for associations with serum uric acid. However, there was no significant association between blood lead and hyperuricemia in males.

Animal studies have shown that serum uric acid was increased in the lead-exposed rats, considered to be a result of renal toxicity of lead [19,20,21]. Moreover, extrarenal mechanisms such as lead effects on purine metabolism have also been considered [22]. However, most of these models were under a short-term [19,20,22] or a single acute lead exposure [21], which overlooked the body's adaptive ability in the long-term. Human studies conducted by clinical researchers have also reported an association between blood lead and serum uric acid, but the results remained controversial. Khan *et al*. [23] found that the serum uric acid level was significantly increased in lead-exposed workers. Wang *et al*. [12] also demonstrated a positive correlation between blood lead and serum uric acid (β = 0.0085, *p* = 0.024). Consistently, the significant effects of blood lead on serum uric acid were shown in several studies [13,14,24]. However, unchanged serum uric acid in lead-exposed workers was reported by Omae *et al*. [15] Weaver *et al*. [16] found no association between blood lead and serum uric acid in Korean Lead Workers as well, after adjusting for age, gender, BMI, and alcohol use. Therefore, large-scale epidemiological studies are needed for verifying the relationship between blood lead and serum uric acid. 

Most of the previous studies have focused on the occupational groups, who are suffering more serious lead pollution but less years of exposure. However, our study confirmed a significant linear relationship between blood lead and serum uric acid level on the general population, who perhaps suffering continuous lead pollution from birth with a relative low level of lead exposure. Many regions of developing countries like China, India [25] have suffered continuous lead exposure for decades and will still suffer lead exposure for a long time. Our results accurately represent the effects of lead exposure on populations in these regions, and are more meaningful for these regions to formulate public health policies. 

A national cross-sectional survey reported that the prevalence of hyperuricemia among Chinese adults was much higher in males (9.9%) than in females (7.0%) [17]. In addition, the gender disparity was more obvious in some other studies [18,34,35]. However, the results of our study showed that the prevalence of hyperuricemia in a lead-polluted area was higher in females (8.1%) than in males (6.3%), although the difference was not statistically significant (*p* = 0.106). In our study, the regression coefficient of blood lead with serum uric acid was higher in females (β = 27.374) than in males (β = 18.575). Interestingly, only female participants in the highest blood lead quartiles had significantly higher prevalence of hyperuricemia compared to the lowest blood lead quartile (OR = 2.190, *p* = 0.025). Lead exposure was not significantly associated with a higher prevalence of hyperuricemia in males, perhaps due to the weaker association between blood lead and serum uric acid in males. The reason why blood lead had a greater impact in females still remains unclear. Gender differences in genetic susceptibility to lead exposure and additional endogenous lead exposure occurs at menopause and the following years may play a role [36]. Further studies are needed to explore and confirm this gender-based divergence. 

There were some limitations in our study. First, we could not infer any causal relationships between blood lead and serum uric acid levels in this cross-sectional study. Second, only single serum uric acid measurement was available, which was not as desirable as using the mean of several measurements. Third, dietary factors were known to be associated with serum uric acid levels, but we did not include them as confounding variables. However, dietary habits of populations living in an inland area of China were relatively similar.

## 5. Conclusions 

In conclusion, continuous lead exposure has an independent impact on serum uric acid in both males and females, although this impact is more pronounced in females than in males. Lead exposure is significantly associated with hyperuricemia for females but not for males. Continued efforts should be devoted to controlling serum uric acid in populations living in a lead-polluted area, particularly among females. 
